# Separation of overlapping dental arch objects using digital records of illuminated plaster casts

**DOI:** 10.1186/s12938-015-0066-9

**Published:** 2015-07-11

**Authors:** Mohammadreza Yadollahi, Aleš Procházka, Magdaléna Kašparová, Oldřich Vyšata, Vladimír Mařík

**Affiliations:** Department of Computing and Control Engineering, University of Chemistry and Technology in Prague, Technická 5, 166 28 Prague 6, Czech Republic; Department of Paediatric Stomatology, The Second Medical Faculty, Charles University, V Úvalu 84, 150 06 Prague 5, Czech Republic; Department of Neurology, Charles University, Sokolská 581, 500 05 Hradec Králové, Czech Republic; Czech Institute of Informatics, Robotics and Cybernetics, Czech Technical University, Zikova 1903/4, 166 36 Prague 6, Czech Republic

**Keywords:** Orthodontic digital modelling, Illumination, Image segmentation, Region growing method, Hough transform, Dental arch, Computational intelligence

## Abstract

**Background:**

Plaster casts of individual patients are important for orthodontic specialists during the treatment process and their analysis is still a standard diagnostical tool. But the growing capabilities of information technology enable their replacement by digital models obtained by complex scanning systems.

**Method:**

This paper presents the possibility of using a digital camera as a simple instrument to obtain the set of digital images for analysis and evaluation of the treatment using appropriate mathematical tools of image processing. The methods studied in this paper include the segmentation of overlapping dental bodies and the use of different illumination sources to increase the reliability of the separation process. The circular Hough transform, region growing with multiple seed points, and the convex hull detection method are applied to the segmentation of orthodontic plaster cast images to identify dental arch objects and their sizes.

**Results:**

The proposed algorithm presents the methodology of improving the accuracy of segmentation of dental arch components using combined illumination sources. Dental arch parameters and distances between the canines and premolars for different segmentation methods were used as a measure to compare the results obtained.

**Conclusion:**

A new method of segmentation of overlapping dental arch components using digital records of illuminated plaster casts provides information with the precision required for orthodontic treatment. The distance between corresponding teeth was evaluated with a mean error of 1.38% and the Dice similarity coefficient of the evaluated dental bodies boundaries reached 0.9436 with a false positive rate $FPR=0.0381$ and false negative rate $FNR=0.0728$.

## Background

In the fields of orthodontics and dentofacial orthopaedics, the optimal timing with regard to the patient’s age and skeletal maturity is just as important as identification of the most appropriate treatment process. Depending on his or her actual age, it is critical to identify the growth periods that provide an opportunity to correct the existing dentofacial irregularities while minimizing the potential risks of the orthopedics intervention using dental arch analysis. Multidisciplinary dental care [1] and therapy requires close collaboration between the different medical specialists and professionals, including the surgeon, orthodontist, and prosthodontist.

Although dental casts have been used for diagnosis and treatment planning [2, 3] in various fields of dentistry for a long time, the digitalization of plaster casts gives the opportunity for their better analysis, enhancement and classification. Digital models, unlike traditional dental casts, allow sharing the models with other specialists during the therapy and treatment [4] and eliminating the challenges related to their storage and transfer. Application of computer science and digital technologies, such as digital data acquisition, virtual models, computed tomography, and video image processing [5], help in the diagnosis and treatment of orthodontic patients and in surgery as well. Digital data can be further improved by the study of their evolution by registration methods in selected regions of interest.

**Figure 1:**
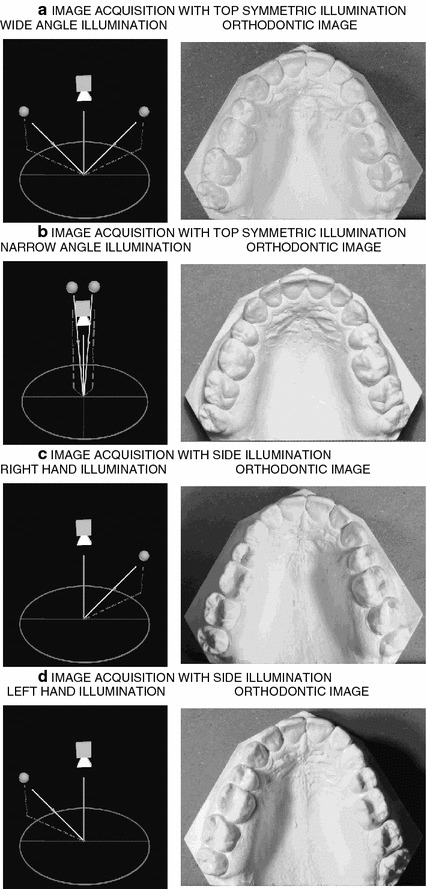
Digital data acquisition of the orthodontic plaster cast by a digital camera for **a**,** b** top illumination, **c** right hand side illumination, and **d** left hand side illumination.

The aim of this paper is to analyse digitized dental plaster casts [6–9] by a combination of several data sets acquired with different side illumination sources. Figure 1 shows digital images of a standard plaster cast obtained with different positions of the illumination sources during their acquisition. The paper presents their analysis to study the evolution of the dental arch during the orthodontic treatment [10] using digital processing and segmentation techniques.

**Figure 2:**
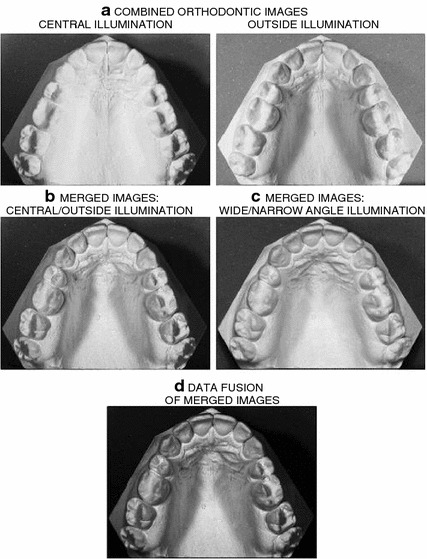
Image enhancement including **a** combination of images with side illumination, **b** merging of images with central/outside illumination, **c** merging of images with top wide/narrow angle illumination, and** d** fusion of merged images resulting in the final image.

**Figure 3:**
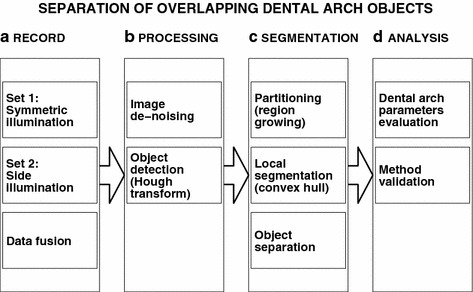
Block diagram of the proposed methodology including** a** image acquisition and data fusion,** b** image de-noising and the use of Hough transform for detection of individual objects,** c** segmentation based on the region growing method, and** d** evaluation of dental arch parameters.

## Digital data acquisition

For more than a century, traditional film radiographs were used at most dental clinics before digital dental radiography was firstly introduced [11] in the late 1980s. The traditional film radiography has been replaced by digital dental radiography as it has more advantages, such as clinical accuracy, better resolution, reduced radiation exposure of the patient, easy storage, communication and transfer of data.

The latest developments in computer technology enable creating electronic tools that can benefit many areas of medicine, surgery and dentistry [12–15]. Imaging technologies in two or three dimensions have become currently applied tools at most health clinics.

**Figure 4:**
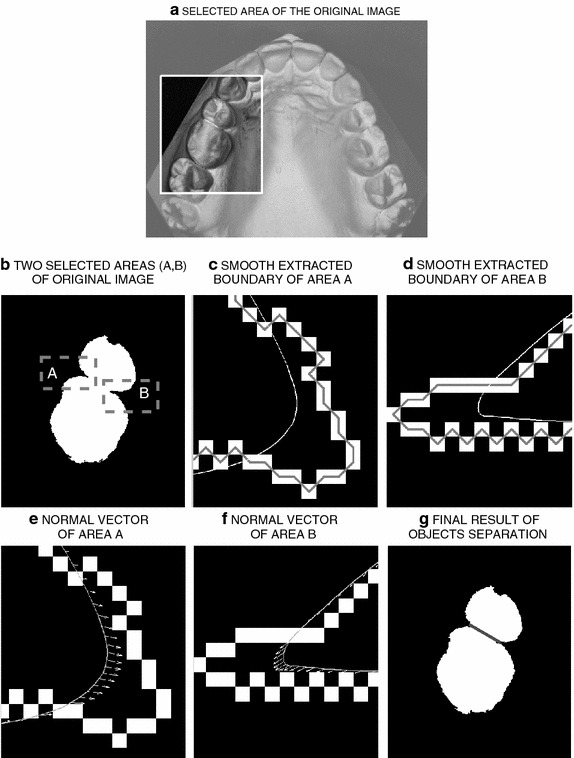
Identification steps of common boundary detection presenting** a** selected area of the original image,** b** dental subimage with two connected neighbouring regions,** c**, **d** details of areas (*A*) and (*B*) with smoothed boundaries,** e**,** f** vectors related to the second derivative of the boundaries showing local convexity in areas (*A*) and (*B*), and** g** resulting segmentation.

**Figure 5:**
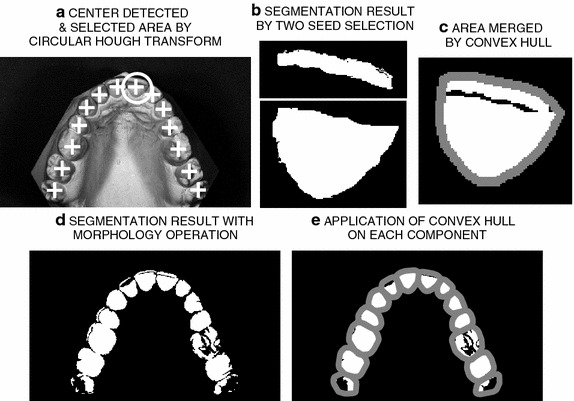
Orthodontic image segmentation presenting** a** the circular Hough transform,** b** segmentation results using the region-growing method (multiple seed points),** c** separate areas merged by the convex hull,** d** segmentation results for the whole dental arch, and** e** convex hull application for each component.

**Figure 6:**
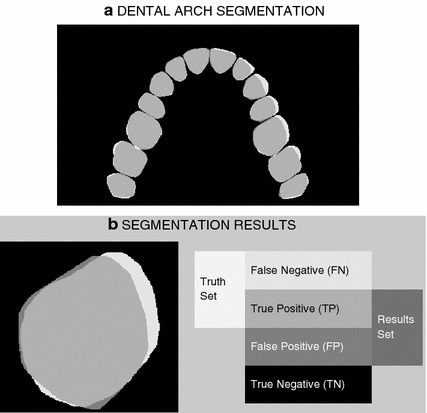
The results of the proposed segmentation process presenting** a** evaluated and real object boundaries for the dental arch and** b** selected tooth (R5) boundaries with false/true positive and false/true negative regions.

Devices for the digital imaging of dental casts include 3D scanners, 3D printers, and digital cameras in general. For the purpose of this paper, the 2D digital images of a standard plaster cast were acquired by a digital camera using different directions of illumination sources to obtain images with different shadow sizes related to the shape of the plaster cast.

The experimental environment included the camera placed at a fixed position above the observed orthodontic plaster cast. The source of illumination was installed at the side of the plaster cast to obtain an image with a shadow and reflection to improve the location of the boundary of the object. Instead of using a single image, a series of images was acquired and combined for the following segmentation process. The location of the illumination source was defined by its azimuth ($\varphi$) and its elevation ($\theta$) angle and a symmetric kind of illumination was selected. The illumination angles were selected experimentally to produce different shadows of the object. A digital camera using a CMOS sensor was used to obtain the set of grey-level images ${\mathbf{A}}_i(\varphi _i,\theta _i)$, $i=1,2, \ldots$ as a function of the illumination source position [16]. Figure 1 displays selected images obtained.

Combination of the images acquired with the different side illuminations was used to improve the contours of the separate orthodontic plaster cast objects. The proposed method combined four grey-scale images obtained for the left, right and two top illuminations for further processing. Identical images acquired with the left-hand side and right-hand side illumination were divided into two parts using the central vertical axis. The combination of the separated subimages formed two new images having outside and central illumination. The two images illuminated from the top were multiplied pixel by pixel to form one output image. The resulting combined image was then obtained after the pixel by pixel mutliplication of all these images. Figure 2 illustrates the entire process of image combination.

For further image analysis, the quality of the image needs to be improved by digital signal processing tools, including digital filters to remove blurring, to increase the contrast, and to achieve higher accuracy in the separation of the image components.

## Methods of orthodontic data processing

Figure 3 presents the proposed methodology that consists of (a) image acquisition using different kinds of illumination and data fusion, (b) image processing including its de-noising and the use of Hough transform for detection of individual objects, (c) segmentation based on the region growing method followed by local segmentation and object separation, and (d) estimation of dental arch parameters.

### Image de-noising

Image de-noising, representing an important concept in image processing, is one of the main tools for the enhancement of (orthodontic) image quality. The noise of the image negatively affects the quality of the image, changing the true grey-level values of each its pixel. Such noise can be caused by a number of factors, including image acquisition conditions, illumination level, positioning of illumination sources, and scene environment.

The initial analysis of the image noise components allows designing the appropriate filter to reduce the noise and to keep the desired information. Noise components of the digital camera (using CCD or CMOS sensors) can be classified into two main categories: the fixed pattern noise caused by sensor non-uniformities and temporal noise. Temporal noise is a non-ideality noise in an image sensor which varies randomly over time. In fact, this type of noise varies from frame to frame and is independent across pixels. The sources of noise related to the camera include photon shot noise, dark current, readout noise, reset noise, and quantization noise.

The Wiener filter was applied as a type of low-pass filter [17] that adapts itself to the local image variance. It allows better smoothing results in case the variance is small owing to statistics estimating the local mean and variance around each pixel. The Wiener filter is especially suitable for reducing speckle, Poisson, and Gaussian noise.

The Wiener filter output ${\mathbf{B}}=\{b(i,j)\}$ using the $R\!\!-\!\!by\!\!-\!\!S$ local neighbourhood $\eta$ of each pixel in the given image ${\mathbf{A}}=\{a(i,j)\}_{i,j=1}^{M,N}$ is estimated by

1 $$\begin{aligned} b(i,j)=\mu +\frac{\sigma ^{2}-\xi ^{2}}{\sigma ^{2}} (a(i,j)-\mu ) \end{aligned}$$

where $\xi ^{2}$ is the image noise variance (or average of all its local variances) and $\mu$, $\sigma$ respectively describe the local mean and variance around each pixel:

2 $$\begin{aligned}\mu &= \frac{1}{R\,S} \sum \limits _{i,j \epsilon \eta } a(i,j), \nonumber \\\sigma ^{2}&=\frac{1}{R\,S} \sum \limits _{i,j \epsilon \eta } a^{2}(i,j)-\mu ^{2}. \end{aligned}$$

The median filter was used in image processing as a robust filter [18, 19] that preserves the edges of an image. It is a nonlinear filter for removing impulsive noise, and replaces the value of one pixel *a*(*i*, *j*) with the median value of all $Q=R\,S$ pixels in its $R\!\!-\!\!by\!\!-\!\!S$ neighbourhood:

3 $$\begin{aligned} b(i,j)= {\left\{ \begin{array}{ll} p_{\frac{Q+1}{2}} &{}\text {if}\,\, Q \hbox { is odd} \\ 0.5\;\left(p_{\frac{Q}{2}}+p_{\frac{Q}{2}+1}\right) &{}\text {if}\,\, Q \hbox { is even} \end{array}\right. } \end{aligned}$$

where $p_{1},p_{2},\ldots,p_{Q}$ represent the intensity values in the $R\!\!-\!\!by\!\!-\!\!S$ neighbourhood of the reference pixel, arranged in either increasing or decreasing order.

### Object detection by the circular Hough transform

The determination of the curvature and location of circular objects in an (orthodontic) image are important tasks [20] in machine intelligence, computer vision, and image analysis [21].

The circular Hough transform used in the present paper obtains satisfactory results in the detection of circle patterns within an image [22] in noisy environments. It transforms the feature points in the image space into the Hough space. In this paper, the circular Hough transform of the Tao Peng algorithm is used. It is is based on the gradient field with an input orthodontic grey-scale image. The proposed algorithm operates without any loop [23], which makes its operation faster but consumes more memory. The proposed algorithm runs with a specified range of radii (minimum to maximum) to be detected in the orthodontic image and with a threshold for the grey-level gradient.

### Image segmentation using region growing

The segmentation [24–26] of an (orthodontic) image can be performed [27] employing different characteristics, which results in the identification of the boundary or region of interest related to an object. Main approaches to image segmentation include: threshold techniques, boundary based methods, region based methods, and hybrid techniques that combine boundary and region criteria [28].

The proposed algorithm is based upon the region growing method using multiple seed points for segmentation of orthodontic images based on partitioning of an image into regions [28–30] using the properties of the image pixels and their distribution. The application of specific preprocessing techniques prior to the region-based method usually improves the results and makes them more reliable. The region-based method clusters similar pixels into a region by taking into account the neighbourhood of each pixel according to selected properties or certain characteristics, including texture, colour or intensity [31].

Pixels that have similar properties form a region and are grouped together. The purpose of image segmentation [32] is to divide the whole image ${\mathbf{A}}=\{a(i,j)\}_{i,j=1}^{M,N}$ into *Q* connected sub-regions ${\mathbf{R}}_{1},\,{\mathbf{R}}_{2},\ldots,{\mathbf{R}}_{Q}$ covering the whole image, which means that $\bigcup _{k=1}^{Q} {\mathbf{R}}_{k}={\mathbf {A}}$ and ${\mathbf{{R}}}_{k}\bigcap {\mathbf{{R}}}_{l}=$ Ø for all $k \ne l.$

The region growing method is initiated with the appropriate selection of a set of seed points. When there exists a priori information about the image properties, such starting points can be defined directly. Otherwise, selected properties should be evaluated for each pixel and after the initial clustering process, seeds can be defined in the centroids of the obtained clusters. The growing starts from the initial seed points and using predefined criteria makes it possible to group pixels with similar properties into larger regions.

The iteration process of the region-growing method can be stopped in case all pixels are distributed into regions according to the predefined criteria but some additional conditions can be added, such as region sizes or their shapes. According to the threshold values selected and the sensitivity, the extracted region may grow over the actual region boundary. The suitable selection of seed points, stopping rules, thresholding, and sensitivity [33] are very important for the efficiency of the whole process. Where the borders of the object are extremely difficult to detect, the result of segmentation by region growing is often very satisfactory [34].

### Detection of image components

The region growing method applied to one object results in several sub-areas. Their merging can be done using computational geometry and detection of a convex hull $C({\mathbf{Z}})$ of an object $\mathbf{Z}$ [35, 36] composed of *T* components in two-dimensional space. The associated morphology algorithm can then be used to define the convex hull by $C({\mathbf{Z}})=\bigcup _{k=1}^{T} {\mathbf{R}}_{k}$ where ${\mathbf{R}}_k$ is the *k*th convex hull component for $k=1,2,\ldots,T$.

The separation of two connected neighbouring regions when their common boundaries were removed during data processing is an important issue in image analysis and machine vision applications. The identifying of a common boundary between two regions or two overlapping objects is usually challenging, as one segment is incorrectly detected by the segmentation process. Several studies and algorithms [37] have been developed to overcome this problem using different methods, including watersheds, the Otsu method, and adaptive thresholding, for the separation of two overlapping objects. Among these methods, watersheds and their modifications are methods commonly used, although for complex areas, watersheds often result in over-segmentation.

In this paper, we propose identifying the common boundaries of two connected neighbouring regions for orthodontic images presented in Figure 4a using mathematical morphology taking into account the geometrical properties of the objects [38, 39] to extract the relevant information about the given bodies in the image. The proposed algorithm consists of the following steps:

- Application of a number of morphological operators, such as dilation performed for boundary extraction, filling the holes to remove unwanted regions in the binary image, and shrinking for reducing the objects on the boundary to a single point. Dilation aims to expand objects in a binary image [40] where the pixels of the objects are expanded to neighbouring pixels. The magnitude of the enlargement of the objects is controlled by different shapes [41] and values as structuring elements.
- The application of boundary tracing of two connected neighbouring regions and smoothing of the traced boundary by a moving average filter. In the binary image, the foreground pixels are labelled by ‘one’ and the background pixels are labelled by ‘zero’ [42] so that in the boundary tracing, the pixels of the foreground are detected.
- Calculation of the second derivative at each point on the smoothed boundary of two connected neighbouring regions.
- Determination of specific zones that contain the intersection points of two connected regions based on the second derivative that are situated inside of the object.
- Evaluation of the absolute extreme of the two zones obtained in the previous step which will mark the position of the intersection points of the two connected regions.

Figure 4c, d show the extraction of the boundary of these regions by morphology methods in areas A and B. White squares present the boundaries of the object smoothed by the moving average filter with resulting curves shown in these subimages as well. Figure 4e, f illustrate smoothing and tracing the boundary to identify the second derivative in selected areas A and B to identify local convexity of object boundaries. The result of the final segmentation of the original image is shown in Figure 4g.

### The proposed methodology

The newly proposed method of dental arch image processing based on separate methods described above consists of the following steps:

1. Image acquisition with the proposed illumination strategy and fusion of image matrices obtained;
2. Wiener and median filtering of image data to reduce their noise components;
3. The use of circular Hough transform to apply local segmentation for individual teeth;
4. Application of the region growing method with multiple seed points to find boundaries of individual sub-images provided by the circular Hough transform;
5. Merging of corresponding sub-areas using computational geometry and convex hull regions to separate overlapping objects as well;
6. Evaluation of dental arch parameters and measures using centers of mass of individual objects detected by the previous segmentation process.

Measures obtained are used for evaluation of the effect of the invasive or non-invasive treatment in stomatology. The segmentation process proposed enables semiautomatic evaluation of mass centers of individual objects and more efficient analysis of location of individual teeth.

## Results

Figure 5 shows the fundamental steps of the complete algorithm for dental arch analysis. In Figure 5a it is possible to see the application of the circular Hough transform for the original image to crop the image to sub-images for feeding to the region growing method applying multiple seed points in Figure 5b. The result of the convex hull method used to merge the regions resulting from the region growing method for a selected object is displayed in Figure 5c. Figure 5d, e show segmentation results for the whole dental arch.

Figure 6 presents a comparison of the dental arch segments obtained by the proposed process and the boundaries of real objects, in order to numerically evaluate the results.

Results of the proposed segmentation process were further analysed by a confusion analysis [43]. Image pixels inside real boundaries (positive/truth set) and those inside boundaries resulting from the proposed segmentation method (results set) specified in Figure 6 can be divided into four categories [44, 45]: true positive (*TP*) and false negative (*FN*) pixels in the positive set, and true negative (*TN*) and false positive (*FP*) pixels in the negative set (outside the positive set). The number of pixels belonging to these regions define the following:

- Sensitivity as the true-positive rate of the correct positive classification in the positive set
  4 $$\begin{aligned} TPR=\frac{TP}{TP+FN}; \end{aligned}$$
- Specificity as the true-negative rate of the correct negative classification in the negative set
  5 $$\begin{aligned} TNR=\frac{TN}{FP+TN}; \end{aligned}$$
- Probabilities of false classifications in the positive set (false-negative rate) and negative set (false-positive rate)
  6 $$\begin{aligned} FNR&= \frac{FN}{TP+FN}, \end{aligned}$$
  7 $$\begin{aligned} FPR= \frac{FP}{FP+TN}; \end{aligned}$$
- Accuracy as the measure of correct classification
  8 $$\begin{aligned} Accuracy=\frac{TP+TN}{TP+TN+FP+FN}; \end{aligned}$$
- Jaccard similarity index and Dice coefficient
  9 $$\begin{aligned} JaccInd= \frac{TP}{FP+TP+FN}, \end{aligned}$$
  10 $$\begin{aligned} DiceCoef= \frac{2\;\;TP}{(FP+TP)+(TP+FN)}; \end{aligned}$$
  used to evaluate set agreements and the results of the segmentation process [46–48] with their values inside the range [0, 1] and individual coefficients close to one for a complete correspondence between evaluated and real object boundaries.

The numerical results presented in Table 1 include the Jaccard similarity index, Dice coefficient, accuracy, and probabilities of false classifications (false-negative rate, false-positive rate) evaluated for separate dental bodies. The results confirm a good correspondence between evaluated and real object boundaries with high similarity indices and low false negative and false positive rates.

**Table 1:** Evaluation of the proposed segmentation by the Jaccard similarity index and Dice coefficient showing their largest values in italics

	Similarity measures				
	Jaccard	Dice	Accuracy	FPR	FNR
Teeth right-side					
1R	0.9330	0.9653	0.9993	0.0627	0.0086
2R	0.9211	0.9589	0.9994	0.0649	0.0192
3R	*0.9606*	*0.9799*	*0.9996*	0.0285	0.0120
4R	0.9344	0.9661	0.9994	0.0512	0.0178
5R	0.9466	0.9726	0.9989	0.0363	0.0190
6R	0.9217	0.9593	0.9986	0.0308	0.0499
7R	0.8933	0.9436	0.9988	0.0198	0.0891
Teeth left-side					
1L	0.8786	0.9354	*0.9998*	0.0611	0.0677
2L	0.8224	0.9025	*0.9998*	0.0146	0.1656
3L	0.7285	0.8429	0.9974	0.0654	0.2238
4L	0.8360	0.9106	0.9983	0.0455	0.1261
5L	0.8593	0.9243	0.9971	0.0427	0.1040
6L	0.8964	0.9454	0.9983	0.0248	0.0813
7L	*0.8986*	*0.9466*	0.9990	0.0309	0.0737

*FPR* false positive rate,* FNR* false negative rate.

Similarity measures evaluated for the whole dental arch include the Jaccard index $JaccInd=0.8931$ and Dice coefficient $DiceCoef=0.9436$ and $Accuracy=0.9828.$ Regions incorrectly classified are represented by the false positive rate $FPR=0.0381$ and false negative rate $FNR=0.0728.$

The comparison of selected measures obtained by manual and digital measurements is summarized in Table 2. The distance between corresponding teeth was evaluated with a mean error of 1.38%.

**Table 2:** Distances (mm) between mass centres of symmetric regions for manual and proposed segmentation

	Distances (mm)		Error	
	Manual seg.	Proposed seg.	mm	%
Teeth number				
1-1	32.68	32.14	0.54	1.65
2-2	85.86	84.69	1.17	1.36
3-3	124.15	121.38	2.77	2.23
4-4	147.82	145.39	2.43	1.64
5-5	168.63	166.44	2.19	1.30
6-6	206.01	203.77	2.24	1.09
7-7	227.72	226.87	0.85	0.37

## Conclusion

This paper presented an innovative approach to the segmentation of orthodontic plaster cast images. The proposed method is based on processing the image constructed from separate images acquired with different illumination sources reflecting different edges of the object. The combined image with its increased contrast and enhanced object boundaries is then used for the detection of separate object.

The results of segmentation of a digital image of the orthodontic plaster cast by the method proposed in this paper show that the convex hull followed by the separation of two connected objects form effective complementary techniques to improve the segmentation by the region growing method.

The illumination from different sides highlights shadows of the object, converting each region into several sub-regions: hence, region growing, based on the application of multiple seed points, is a suitable tool to extract individual bodies. However, the method (1) does not produce satisfactory results in the common boundary of two regions that have similar properties and (2) the identified sub-regions related to the same region are not always recognized as one region.

The final evaluation of the segmentation process points to the efficiency of the proposed method with a Dice similarity coefficient of 0.9436 and a mean error of real and estimated distances between corresponding teeth of 1.38%.

Further studies will be devoted to further more sophisticated methods based upon three-dimensional convex hulls, used for the separation of individual bodies, as well as to a more detailed analysis of the shapes of the separate dental arch components.
